# The ^18^F-FDG PET/CT response to radiotherapy for patients with spinal metastasis correlated with the clinical outcomes

**DOI:** 10.1371/journal.pone.0204918

**Published:** 2018-09-28

**Authors:** Jinhyun Choi, Jun Won Kim, Tae Joo Jeon, Ik Jae Lee

**Affiliations:** 1 Department of Radiation Oncology, Gangnam Severance Hospital, Yonsei University College of Medicine, Seoul, Korea; 2 Department of Radiation Oncology, Jeju National University Hospital, Jeju National University School of Medicine, Jeju, Korea; 3 Department of Nuclear Medicine, Gangnam Severance Hospital, Yonsei University College of Medicine, Seoul, Korea; Northwestern University Feinberg School of Medicine, UNITED STATES

## Abstract

**Purpose:**

To evaluate the potential role of ^18^F-fluorodeoxyglucose-positron emission tomography/computerized tomography (FDG-PET/CT) for predicting treatment response after radiotherapy (RT) in patients with spinal metastases.

**Materials and methods:**

A retrospective analysis was performed of 42 patients with spinal metastases who received RT from January 2010 to December 2014. All patients underwent FDG-PET/CT before and after treatment. Changes in metabolic responses, expressed as the maximum, mean, peak standardized uptake values (SUVmax, SUVmean, SUVpeak), metabolic tumor volume (MTV) and total lesion glycolysis (TLG) were analyzed to determine their association with clinical outcomes.

**Results:**

The median age at the time of spinal metastasis diagnosis was 58 years. Median progression-free survival (PFS) and overall survival after RT were 15 months and 22.4 months, respectively. RT produced a significant decrease in SUVmean (2.27 to 1.41), SUVmax (6.87 to 2.99), SUVpeak (5.75 to 2.33) and TLG (52.84 to 24.17) when compared with the baseline values (p<0.001). The mean pain score decreased from 3.86 before RT to 0.79 after RT (p<0.001). There were significant linear relationships between maximum SUV and pain scores at baseline (r = 0.321, p = 0.038) and after treatment (r = 0.369, p = 0.016) as well as TLG at baseline (r = 0.428, p = 0.005) and after treatment (r = 0.403, p = 0.009). Local progression after treatment was identified in 12 patients (28.6%). Univariate analyses showed that >70% reduction in maximum SUV after treatment was independently associated with good PFS (p = 0.036).

**Conclusions:**

RT is an effective treatment for patients with spinal metastases, and there were significant changes in PET parameters compared with baseline. The metabolic response measured by SUV and TLG changes in FDG-PET/CT correlated with the clinical outcomes, especially with shorter PFS in patients who had higher residual maximum SUV after treatment.

## Introduction

The spine is the most frequent bone metastatic site, and spinal metastases have been reported in approximately 5% to 14% of all cancer patients [1]. Spinal metastases often cause severe morbidity and adversely affect quality of life because of pain, pathologic fractures, and spinal cord compression [2, 3]. Treatment options for spinal metastases include surgery, radiotherapy (RT), and systemic therapy, such as bisphosphonates; the goal of these modalities is palliation of pain, maintenance of spinal stability, prevention of neurologic deficit, and slowing of local disease progression [4, 5]. RT, the most common strategy for spinal metastases, has been reported to provide significant palliation of pain in approximately 60% to 90% of patients, with up to 33% of individuals achieving complete pain response at the irradiated site [4, 6, 7]. As the life expectancy of patients with successful treatment of spinal metastases is increasing, proper assessment of treatment response is essential for making correct treatment decisions and improving outcomes.

Imaging of tumor metabolism with ^18^F-fluorodeoxyglucose-positron emission tomography/computerized tomography (FDG-PET/CT) has been widely applied in the evaluation of various malignancies. Changes in tumor glucose utilization provide quantitative and qualitative functional information [8]. ^18^F-FDG-PET/CT has been proven useful in assessing prognosis and monitoring treatment outcomes, and a number of studies have shown that PET parameters correlate with radiologic response in several tumor types [9–11]. However, few studies have reported the prediction power of ^18^F-FDG-PET/CT in patients who received RT for spinal metastases [12, 13].

The aim of this study is to assess the role of metabolic monitoring by ^18^F-FDG-PET/CT for predicting treatment response after RT in patients with spinal metastases. We evaluated whether changes in PET parameters including the standardized uptake value (SUV) and total lesion glycolysis (TLG) after treatment are correlated with clinical results.

## Materials and methods

### Patients

A total of 42 patients with spinal metastases who received RT from January 2010 to December 2014 were evaluated. We retrospectively reviewed the medical records of these patients and collected data regarding their sex, age, clinical presentation, primary cancer, radiologic features, presence of metastases at other sites, type of treatment, and clinical course. All patients underwent physical examination including neurologic assessments at baseline and 1, 3, and 6 months after RT. Pain intensity at the treatment site was evaluated every assessment day using the Brief Pain Inventory, which assesses pain at its “worst,” “least,” “average,” and “now” (current pain). In our institution, “worst” was used to represent pain severity experienced for previous 24 hours by the numeric rating scale, in which 0 = no pain to 10 = worst pain imaginable. Characteristics of the spinal metastases, such as the involved segment or the presence and location of a pathologic fracture or cord compression, were obtained using imaging studies, including plain radiography, computed tomography (CT), and magnetic resonance imaging (MRI). All patients underwent FDG-PET/CT prior to treatment and after treatment. This Institutional Review Board of the Severance Hospital, Korea (IRB No. 3-2011-0281) approved this retrospective study in accordance with ethical guidelines and the Declaration of Helsinki. The consent was not necessary, because patient records and information were anonymized and de-identified prior to analysis.

### Radiotherapy and response assessment by FDG-PET/CT

Each patient underwent CT-based simulation, and structures were manually contoured on CT scan slices. The planning target volume included the specific vertebral body affected, with 0 to 1 cm extension around the clinical target volume. RT was given once per day with 3-dimensional conformal RT, intensity-modulated RT, or stereotactic body RT. Patients received radiation at a dose of 20–54 Gy in 3–30 fractions. The median total dose, which was converted to the biologically effective dose (BED), was 50 Gy_10_.

All ^18^F-FDG-PET/CT data obtained before and after treatment for each patient were transferred to MIMvista (MIM Software, Inc., USA) for objective comparisons. A region of interest was drawn with a 5-cm diameter where the lesion appeared to have the largest uptake at baseline. The same site was identified on the post-treatment image, even when there was a complete response. To evaluate the metabolic response, the SUVmean, SUVmax, maximum average SUV within a 1-cm^3^ spherical volume (SUVpeak), metabolic tumor volume (MTV) and TLG were measured at the region of interest. We defined MTV as total tumor volume with a SUV of 2.5 or greater, and TLG as the product of SUVmean and MTV. The percentage reduction in SUVmax between before and after RT was calculated as follows: % reduction = 100 × (pre-RT SUVmax − post-RT SUVmax)/pre-RT SUVmax.

### Statistical analysis

The primary endpoint was the response rate of patients who had decreased SUV values (determined by ^18^F-FDG-PET/CT) after treatment, compared to baseline. Differences between pre- and post-treatment values of PET parameters were compared using the paired t-test. Based on the PET Response Criteria in Solid Tumors (PERCIST) criteria [14], complete metabolic response (CMR) was defined as normalization of all lesions (target and non-target), so that the SUV corrected for the lean body mass (SUL) is less than mean liver SUL and equal to SUL of the normal surrounding tissue. Partial metabolic response (PMR) was defined as > 30% decrease in SUL peak with a minimum 0.8 unit decrease. Progressive metabolic disease (PMD) was defined as a > 30% increase in SUL peak with a minimum 0.8 unit increase in SUL peak, or the presence of new lesions. Stable metabolic disease (SMD) was disease that did not meet the CMR, PMR, or PMD criteria. Patients with a CMR or PMR were defined as responders, and patients with SMD or PMD as non-responders. This study was designed to assess whether metabolic monitoring with FDG-PET/CT after RT could predict the time to local progression or death. Recurrence or progression at the treatment site defined with CT or MRI-based follow-up imaging studies. The imaging findings compared to previous studies had reviewed by radiologists. In case of new lesions developed other than treatment site, we excluded them from analyzing progression-free survival (PFS) in this study. PFS and overall survival (OS) were measured from the date of starting RT to the date of documented progression, death, or last follow-up. Survival data were analyzed using the Kaplan-Meier method, and comparisons performed using a two-sided log-rank test. The Spearman correlation test was used to determine the association between PET parameters and pain scores. Statistical analyses were performed with SPSS version 20.0 (SPSS Inc., Chicago, USA). A p-value ≤ 0.05 was considered statistically significant.

## Results

### Clinical characteristics

The patients’ median age at the time of spinal metastasis diagnosis was 58 years (range, 24–80 years). Overall, 69% of patients (n = 29) were female, and 54.8% (n = 23) had distant metastasis in addition to the spinal involvement. The most common primary cancer was breast cancer (n = 18), followed by hepatobiliary cancer (n = 10). The treated segment was located in the thoracic spine in 27 patients, lumbar spine in 23 patients, and cervical spine in eight patients. Cord compression on imaging studies was present in 19% of patients. The most frequent extraosseous organ system with a metastasis was the lung, and control of the distant metastases was maintained in 43% of patients. The mean pain score prior to treatment was 3.86 (range, 0–10). Detailed information regarding patient characteristics is presented in Table 1.

**Table 1:** Baseline characteristics and treatment of the 42 patients.

Characteristic	Number of patients, median, or mean	Percentage (%) or range
**Age, years**		
** Median (range)**	58	24–80
**Sex**		
** Male**	13	31
** Female**	29	69
**Primary cancer**		
** Breast**	18	42.9
** Hepatobiliary**	10	23.8
** Lung**	5	11.9
** Head and neck**	4	9.5
** Others**	5	11.9
**Treatment segment**		
** Cervical spine**	8	13.8
** Thoracic spine**	27	46.6
** Lumbar spine**	23	39.6
**Cord compression**		
** Yes**	8	19
** No**	34	81
**RT technique**		
** 3D-CRT**	11	26.2
** IMRT**	23	54.8
** SBRT**	8	19
**Chemotherapy after RT**		
** Yes**	23	54.8
** No**	19	45.2
**Other site metastasis**		
** Yes**	23	54.8
** No**	19	45.2
**Baseline pain score**		
** Mean (range)**	3.86	0–10

RT, radiotherapy; 3D-CRT, 3-dimensional conformal radiotherapy; IMRT, intensity-modulated radiotherapy; SBRT, stereotactic radiotherapy

### Measurement of therapeutic response by FDG-PET

**Fig 1:**
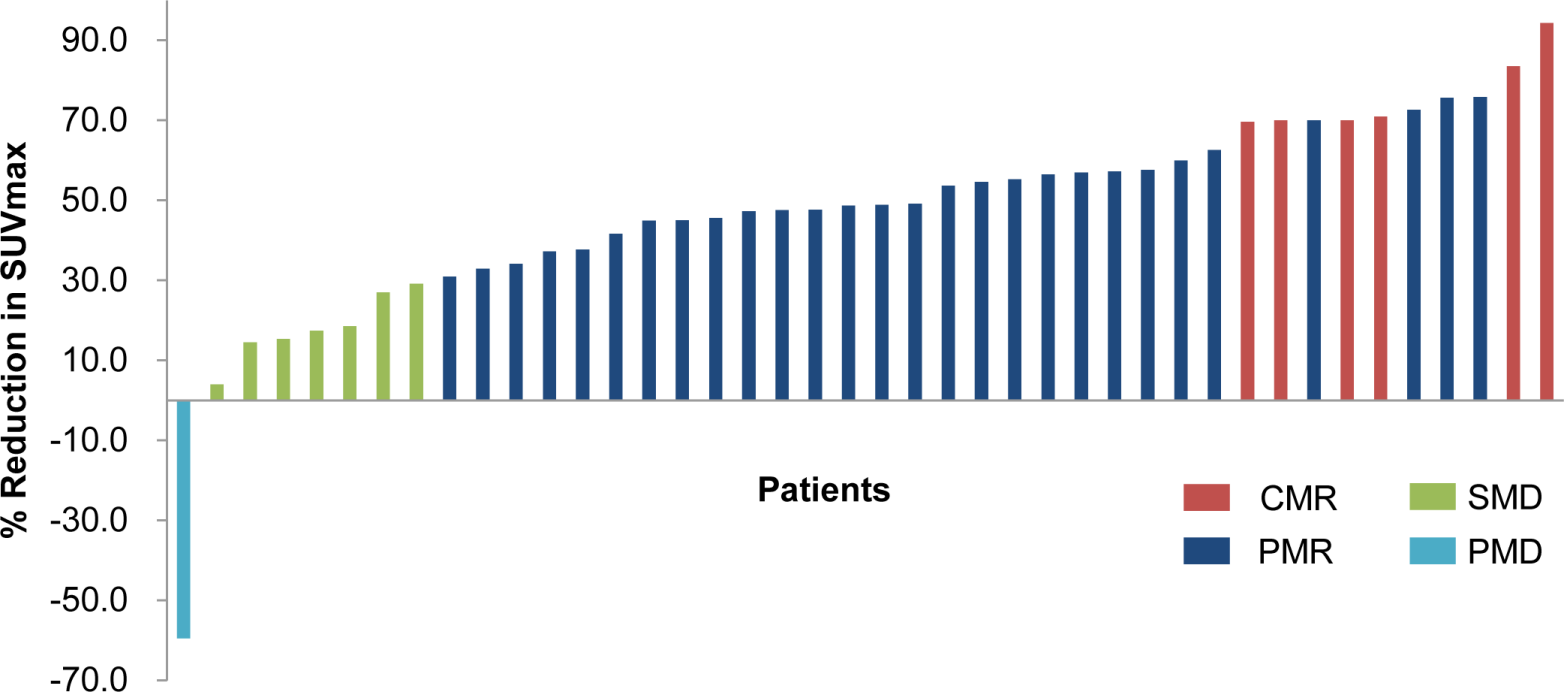
Metabolic response of the 42 patients. Based on the PERCIST criteria, there are some mismatches between metabolic response and SUV reduction in case of an initially low SUV value. One patient with a CMR showed less than 70% reduction SUVmax because the initial value was about 5.

**Fig 2:**
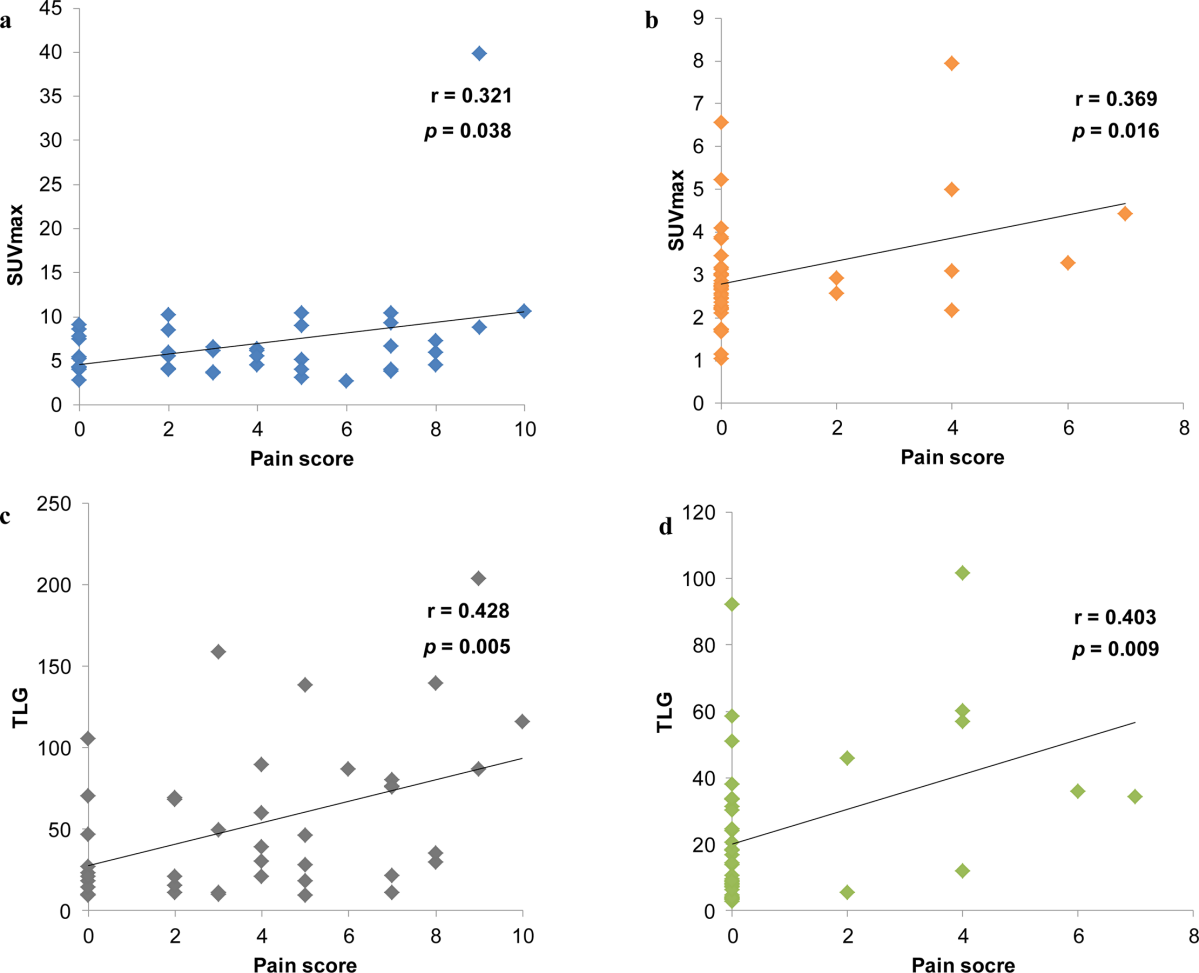
**The relationship between SUVmax and pain scores (a, b), TLG and pain scores (c, d).** (a) at baseline (r = 0.321, p = 0.038), (b) after treatment (r = 0.369, p = 0.016), (c) at baseline (r = 0.428, p = 0.005), and (d) after treatment (r = 0.403, p = 0.009).

RT resulted in a significant decrease in all PET parameters except MTV when compared with baseline values (Table 2). The SUVmax and SUVmean (mean ± standard deviation) were 6.87 ± 0.88 and 2.27 ± 0.2 at baseline, respectively, 2.99 ± 0.2 and 1.41 ± 0.07 after treatment, respectively (p < 0.001). The TLG (mean ± standard deviation) was 52.84 ± 7.19 at baseline and 24.17 ± 3.67 after treatment (p < 0.001). According to the PERCIST criteria using the change in SUV from baseline to after treatment, FDG-PET revealed a PMR in 28 patients (66.7%), SMD in seven patients (16.7%), and PMD in one patient (2.3%). CMR was observed in six patients (14.3%; Fig 1). Overall, 81% of patients were responders, with a metabolic tumor response (CMR or PMR). As shown in Fig 2, there was a significant linear relationship between the SUVmax and pain scores at baseline (r = 0.321, p = 0.038) and after treatment (r = 0.369, p = 0.016) as well as TLG at baseline (r = 0.428, p = 0.005) and after treatment (r = 0.403, p = 0.009). The mean pain score decreased from 3.86 before treatment to 0.79 after treatment (p < 0.001). No significant radiation dose effect was observed after analysis on pain and PET response according to BED.

**Table 2:** ^18^F-fluorodeoxyglucose-positron emission tomography parameters at baseline and after treatment.

Parameter	Pre-treatment		Post-treatment		*p*
	Mean	SD	Mean	SD	
**SUVmax**	6.87	0.88	2.99	0.20	<0.001
**SUVmean**	2.27	0.20	1.41	0.07	<0.001
**SUVpeak**	5.75	0.73	2.33	0.16	<0.001
**MTV**	12.06	1.55	12.04	1.48	0.976
**TLG**	52.84	7.19	24.17	3.67	<0.001

SUV, standardized uptake value; MTV, metabolic tumor volume; TLG, total lesion glycolysis

Data are mean ± standard deviation (SD).

### Prediction of progression-free survival

**Fig 3:**
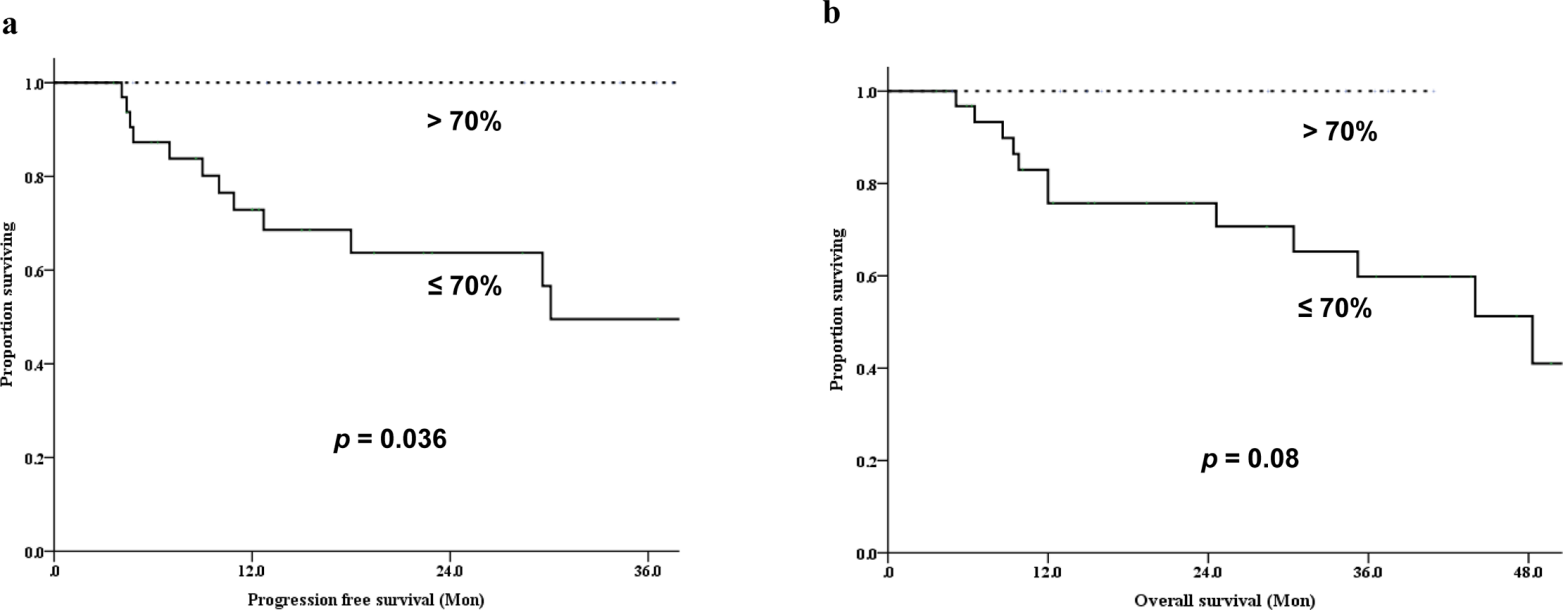
Kaplan-Meier survival curves for patients included in this study. (a) Progression-free survival and (b) overall survival for patients with more than 70% reduction in maximum standardized uptake values versus those with less than 70% reduction.

The median PFS and OS after the start of RT were 15 months (range, 3.6–56.6 months) and 22.4 months (range, 3.6–61.3 months), respectively. Univariate analysis was performed using the variables listed in Table 3, which have been previously suggested to be associated with PFS. Only a reduction in SUVmax was identified as a potential predictor of local progression. Patients with more than 70% reduction in SUVmax between pre- and post-treatment PET studies had better PFS (p = 0.036). There was a trend toward shorter survival in patients with a higher residual SUVmax after treatment (p = 0.08; Fig 3).

**Table 3:** Univariate analysis of variables associated with survival in patients with spinal metastasis.

Variable		2-year PFS		2-year OS	
	No.	%	p	%	p
**Age**			0.468		0.355
** ≤55**	18	72.9		73.3	
** >55**	24	70.7		86.4	
**Sex**			0.581		0.744
** Male**	13	71.3		91.7	
** Female**	29	72.3		77.8	
**Cord compression**			0.852		0.958
** Yes**	8	75.8		87.5	
** No**	34	70.8		79.5	
**CTx after RT**			0.82		0.828
** Yes**	23	69.9		78.9	
** No**	19	71.9		83.6	
**Other site mets**			0.596		0.173
** Yes**	23	73.7		71.4	
** No**	19	69.8		82.4	
**DM control**			0.319		0.205
** Yes**	18	82.4		94.1	
** No**	24	61		63.6	
**RT dose, BED10**			0.656		0.658
** ≤50 Gy**	22	68.8		85.2	
** >50 Gy**	20	76.7		79.1	
**% of SUVmax reduction**			0.036		0.08
** ≤70**	33	63.7		75.7	
** >70**	9	100		100	
**% of SUVmean reduction**			0.103		0.184
** ≤60**	36	66.9		75	
** >60**	6	100		100	
**% of SUVpeak reduction**			0.109		0.184
** ≤70**	35	67.2		78	
** >70**	7	100		100	
**% of TLG reduction**			0.091		0.15
** ≤75**	34	68.3		76.6	
** >75**	7	100		100	

PFS, progression-free survival; OS, overall survival; RT, radiotherapy; BED, biological equivalent dose; SUVmax, maximum standardized uptake value; SUVmean, mean standardized uptake value; SUVpeak, peak standardized uptake value; TLG, total lesion glycolysis

## Discussion

Spinal metastases are frequently encountered during the course of malignancy, even in cancers with a poor prognosis [15]. In general, patient survival is highly dependent on the primary tumor; a previous study reported that the median OS after RT for bone metastases was 16 months for patients with breast cancer and 9.5 months for patients with prostate cancer [16]. Because of the effectiveness of RT for pain and neurological symptoms, RT remains the cornerstone of treatment of spinal metastases [15, 17]. In the current study, the median OS and local PFS were improved after RT to 22.4 months and 15 months, respectively, despite the patients exhibiting heterogeneous primary tumors. The mean pain score also decreased significantly with RT, from 3.86 to 0.79. Previous studies have suggested the histology of the primary tumor, a good performance status, and the absence of visceral metastases as prognostic factors predicting OS and PFS in patients who received RT for spinal metastasis from various malignancies [18–21]. With advances in oncology and the successful treatment of metastatic spinal cord tumors, we expect patients with spinal metastases to live longer. Consequently, a method is required that can estimate their expected survival and help in the selection of successful treatment.

A variety of approaches to measure response rates and PFS have been developed, including the World Health Organization criteria and the Response Evaluation Criteria in Solid Tumors (RECIST) [22]. The response rate typically refers to how frequently a tumor shrinks anatomically; however, lack of progression may be associated with good improvement in outcomes, even in the absence of major tumor shrinkage [23]. Despite effective treatment, changes in tumor size can be minimal in spinal metastases, as the changes depend on the type of metastasis (e.g., whether it is lytic or sclerotic). To determine whether there is a lack of progression by changes in tumor size requires regular and systematic assessment of tumor burden, and PET characteristics appear more informative than CT or MRI in such cases [24]. To overcome this drawback, the PERCIST criteria that integrate metabolic tumor response (i.e., PET) assessment have been proposed. The PERCIST criteria used in this study include assessing the normal reference tissue values in the liver, determining the SUV in a fixed small region of interest in the most active region of metabolically active tumors, and requiring a 30% or greater decline in SUV for a “response” (PMR or CMR) [22]. However, the PERCIST criteria may be limited when assessing the activity of lesions with an initially low SUV value. As shown in Fig 1, among six patients with a CMR, one patient showed less than 70% reduction in SUVmax because the initial SUVmax value was approximately 5. Indeed, all patients with SMD except one had an initial SUVmax of approximately 3 to 4; it is difficult to expect a major reduction in SUV in these cases. Conversely, even PMR patients showed good PFS when they had more than 70% reduction in SUVmax.

**Fig 4:**
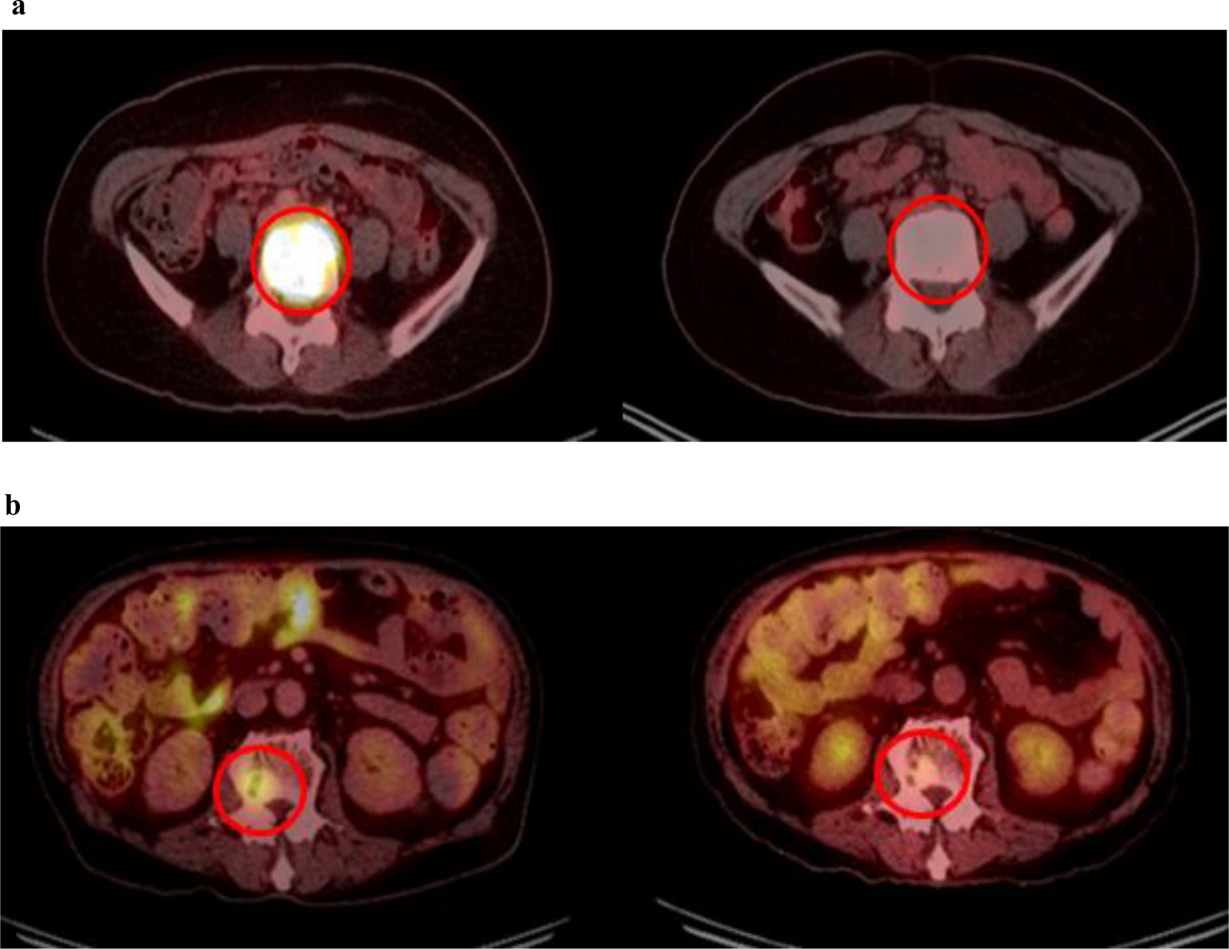
Use of FDG PET/CT to assess treatment response. (a) Complete response after RT with 94.4% reduction (39.79 to 2.23) in maximum standardized uptake values and (b) Partial response with 60% reduction (5.87 to 2.35).

Recently, ^18^F-FDG-PET/CT is receiving attention for predicting the response to radiation, as well as detecting radiographically occult distant metastases [25]. In various tumors, metabolic reduction after treatment has been suggested to be of value in the assessment of treatment response [26, 27]. Tateishi et al. [28] showed that an increased SUV change was a significant predictor of response duration (relative risk, 2.4; p = 0.003). The cut-off points of SUV changes that best predicted clinical outcomes were 60% to 64% in single institutional analyses [9, 29]. Regarding incorporating FDG-PET/CT into RT planning, Tralins et al. [30] applied boost radiation to the increased FDG uptake area, thus achieving dose escalation. In addition, the FDG-PET/CT target volume significantly predicted the time-to-progression of the tumor in their series. The results from these studies thereby support the role of metabolic response to radiation as a predictive marker; however, the role of changes in FDG-PET/CT in patients with spinal metastasis is not well defined. To our knowledge, ours is the first report to determine whether changes in FDG-PET/CT after RT can predict clinical outcomes. Metabolic response to RT, as measured by a change in SUV between baseline and post-treatment, was predictive of PFS. Patients with ≥ 70% reduction in SUVmax demonstrated longer median PFS than those with < 70% reduction (Fig 4). We also found that pain scores at baseline and post-treatment correlated with PET parameters including TLG, and a higher residual SUV was associated with relative resistance to pain relief. However, because MTV is a volume for a specific criterion, the fact that the pre-treatment values ​​did not decrease significantly in this study suggests that the metabolic activity of the part of the lesion is reduced rather than dramatically reduced by RT can do.

Given that our study was retrospective, it has some inherent limitations. It was a single-center study with a small number of patients, and the time interval from completion of RT to the follow-up PET/CT was variable. We included patients who developed distant metastases to organs other than the spine during the course of their disease, as well as patients with uncontrolled disease. The radiation dose can have a considerable effect on patient outcomes. However, because of the heterogeneity of dose profiles and techniques of RT, we did not identify the radiation dose as a significant predictive factor in univariate analysis during the current study. In addition, there was no significance on values of PET parameters between different BED groups. We demonstrated a linear relationship between PET parameters and pain scores, but the inclusion of asymptomatic patients at baseline may have limited the significance of our results. At present, given the cost and mixed diagnostic performance results of PET/CT, there is not enough evidence to support the routine use of PET/CT in monitoring spinal metastases [31]. Therefore, finding the most useful predictors among the various PET parameters, including the SUV cutoff point, remains to be prospectively validated in future studies involving a large number of patients. In addition, further studies are required to determine the optimal time interval between treatment and follow-up PET/CT evaluation, and whether the value of PET may have an impact on therapeutic decision-making. As shown in the correlation analysis, the correlation coefficient of TLG was significantly higher in clinical relevance than the other parameters. This suggests that new parameters such as TLG, which are not traditionally meaningfully analyzed as SUVs, can be a predictor of the patient's prognosis, and the authors believe that future prospective studies will clarify this point.

## Conclusions

RT was an effective treatment for patients with spinal metastases. Pain relief generally occurred after RT, and significant changes in PET parameters compared with baseline were detected. In addition, the metabolic response measured by SUV changes in FDG-PET/CT correlated with clinical outcomes, especially with a shorter PFS in patients who had higher residual SUVmax after treatment. Our results provide evidence that metabolic monitoring with FDG-PET/CT allows the prediction of treatment response after RT in patients with spinal metastases.
